# Supplementary material for: MCMC Methods for Parameter Estimation in ODE Systems for CAR-T Cell Cancer Therapy
Source: Cancers (Basel). 2024 Sep 11;16(18):3132. doi: 10.3390/cancers16183132 (PMC11430073; doi:10.3390/cancers16183132)
Supplement: Supplementary file 1 [file cancers-16-03132-s001.zip › cancers-3110047-supplementary.pdf]

Supplementary Materials: MCMC Methods for Parameter Estimation in ODE Systems for CAR-T Cell Cancer Therapy

Elia Antonini , Gang Mu , Sara Sansaloni-Pastor , Vishal Varma and Ryme Kabak

This Supplementary Material (SM) is organized as follows. Section S1 shows details the existence and uniqueness of the solution of the ODEs system. Section S2 describes in general the bayesian statistical methods used for estimating the parameters. Sections S3 and S4 delve in the description of the MCMC methods and in particular Metropolis-Hastings algorithm. Finally, in the Section S5 the differential evolution approach is shown.

S1. Existence and Uniqueness of the Solution

Here, we will study the solutions of the ODEs system

$$\frac{dC_D}{dt} = -(\beta + \eta)C_D, \tag{S1}$$
$$\frac{dC_T}{dt} = \eta C_D + k(t)F(T)C_T - (\xi + \epsilon + \lambda)C_T + \theta T C_M - \alpha T C_T, \tag{S2}$$
$$\frac{dC_M}{dt} = \epsilon C_T - \theta T C_M - \mu C_M, \tag{S3}$$
$$\frac{dC_E}{dt} = \lambda C_T - \delta C_E, \tag{S4}$$
$$\frac{dT}{dt} = rT(1 - bT) - \gamma f(C_F, T)T, \tag{S5}$$

in which the total number of functional CAR-T cells is:

$$C_F = C_D + C_T,$$

and the functions $k(t)$, $F(T)$, and $f(C_F, T)$ are given by:

$$k(t) = r_{min} + \frac{p_1}{1 + (p_2\, t)^{p_3}}, \tag{S6}$$
$$F(T) = \frac{T}{A + T}, \tag{S7}$$
$$f(C_F, T) = \frac{\frac{C_F}{T}}{\vartheta + \frac{a + C_F}{T}}. \tag{S8}$$

We will use some standard mathematical results.

The theorem of existence and uniqueness for a Cauchy problem, also known as the Picard-Lindelöf theorem [1], the Picard existence theorem, or the Cauchy-Lipschitz theorem, establishes the conditions of existence and uniqueness of the solution of the Cauchy problem

$$\begin{cases} u'(t) = f(t, u(t)), & t \in I; \\ u(t_0) = u_0, \end{cases}$$

Let f be a function defined in a neighborhood of the point $(t_0, u_0) \in \mathbb{R} \times \mathbb{R}^n$ in the form:

$$I \times J = \{(t, u) \in \mathbb{R} \times \mathbb{R}^n : |t - t_0| \leq a, ||u - u_0|| \leq b\}$$

with a, b real positive numbers, and suppose that f is at least of class C^0 in such a neighborhood. It is also assumed that f is Lipschitz continuous with respect to the variable u and uniformly continuous with respect to the variable t:

$$||f(t,u) - f(t,v)|| \leq L||u - v|| \quad \forall t \in I \quad \forall u, v \in J \tag{S9}$$

with L the Lipschitz constant.

Theorem S1. ***(Local Existence and Uniqueness)****[1]*
If f is uniformly continuous with respect to the variable t and satisfies the Lipschitz condition (S9), then there exists $T > 0$, such that the Cauchy's problem

$$\begin{cases} u'(t) = f(t, u(t)), & |t - t_0| < T; \\ u(t_0) = u_0, \end{cases} \tag{S10}$$

has a unique solution

$$u(t) \in C^1((t_0 - T, t_0 + T); J),$$

where

$$J = \{u \in \mathbb{R}^n : ||u - u_0|| < b\}.$$

It is straightforward to observe that the ODEs system (S1)–(S5) under consideration satisfies the local Lipschitz condition as stated in Theorem S1. Consequently, for any given initial condition, the solution exists and is unique locally.

For the global existence we can use the following theorem.

Theorem S2. ***(Global Existence)*** *[2, Corollary 5.1]*
Let be

$$f : \mathbb{R} \times \mathbb{R}^k \longrightarrow \mathbb{R}^k$$

continuous and sublinear, i.e., there are two constants A and B, such that

$$|f(t,u)| \leq A + B|u|$$

for all $(t,u) \in \mathbb{R} \times \mathbb{R}^k$. Then the Cauchy problem

$$\begin{cases} u'(t) = f(t, u(t)), \\ u(t_0) = u_0 \end{cases}$$

has global existence for every initial value.

Our aim is to prove the global existence of the ODEs system (S1)–(S5) for $t \in [0, +\infty]$.

We can start proving the global existence of T. We can divide the equation (S5) using homogeneity:

$$\frac{dT_1}{dt} = rT_1(1 - bT_1), \tag{S11}$$

$$\frac{dT_2}{dt} = \frac{\gamma C_F}{C_F + \vartheta T_2 + a} T_2. \tag{S12}$$

If we prove the global existence of T_1 and T_2 we have done so.

T_1 has a global existence, in fact, it is a well known fact that the analytical solution is

$$T_1(t) = \frac{e^{rt}}{be^{rt} + c_1},$$

where, c_1 is a positive constant since r, b and $T_1(0)$ are positive.

Regarding T_2, since the parameters and T_2 are positive, we have that

$$\left| \frac{\gamma C_F}{C_F + \vartheta T_2 + a} \right| \leq \gamma,$$

so we can conclude using Theorem S2, using sub-linearity.
Then, we can deduce that T is bounded in $[0, +\infty]$. In fact,

$$\frac{dT_1}{dt} = rT_1(1 - bT_1)$$

is an upper solution of T, and as we have seen, T_1 is bounded.

Now, since T is defined and bounded in $[0, +\infty]$, we can deduce that, the problem consists in studying the functions (S7) and (S8). In fact, all the other terms of the system are linear, or multiplied by T, whic is bounded.

So, we can study those specific functions:

- We can start with (S7). We know that all the parameters of the ODEs system are positive for biological reasons, in particular, r_{min}, p_1, p_2 and p_3 are positive. Since, we are interested in $t \in [0, +\infty]$, we have that

$$\left| r_{min} + \frac{p_1}{1 + (p_2\, t)^{p_3}} \right| \leq r_{min} + p_1;$$

- Regarding (S8), since $A > 0$, we can observe that

$$\left| \frac{T}{A + T} \right| \leq \left| \frac{T}{T} \right| \leq 1.$$

So, applying again Theorem S2, we conclude that, if all the parameters are positive (they are for biological reasons), the ODEs system (S1)–(S5) has global existence.

S2. Bayesian Statistical Methods

We present the tools for Bayesian statistical methods. Those tools will help us solve the challenges and advancements in dealing with high-dimensional problems (many parameters). Bayesian analysis, while offering a robust framework for statistical inference, encounters a notable impediment when it comes to computing the posterior distribution for complex models. The integration of high-dimensional functions that arise in the posterior is a task that is not only computationally intensive but also a stumbling block for a broader application. Despite the difficulties, various strategies have been put forth to surmount this barrier, including approximation techniques that avoid the difficult task of direct integration.

We place particular emphasis on Markov Chain Monte Carlo (MCMC) methods, which have revolutionized computational statistics by enabling the sampling from distributions that are challenging to handle with conventional analytical approaches. MCMC methods facilitate the generation of samples by constructing a Markov chain created for the target distribution, using the dependency of each new sample on its predecessor. This creates a powerful tool for statistical inference, allowing for the exploration of the posterior distribution in a manner that was previously unattainable.

S2.0.1. Monte Carlo Integration and Bayesian Inference

Monte Carlo integration is used to calculate the value of an integral for a multivariate function $f(u_1, \ldots, u_d)$ over the domain $\Omega \in \mathbb{R}^d$. The function f is presumed to be square-integrable within this domain.

For convenience, we introduce the notation x to represent a vector in the hypercube, such that $x = (u_1, \ldots, u_d)$, and we write the function at vector x as $f(x) = f(u_1, \ldots, u_d)$. We can estimate the integral of this function using the *Monte Carlo method* [3] as follows:

$$I = \int \partial x\, f(x) = \int \partial^d u\, f(u_1, \ldots, u_d).$$

The estimation is achieved by calculating the average of function values at sampled points:

$$E = \frac{1}{N} \sum_{n=1}^{N} f(x_n),$$

with sample points $x_n = (u_{1,n}, \ldots, u_{d,n})$.

Now, by using the law of large numbers [4], we affirm that as the number of samples N increases indefinitely, our Monte Carlo approximation E asymptotically approaches the exact value of the integral I:

$$\lim_{N \to \infty} \frac{1}{N} \sum_{n=1}^{N} f(x_n) = I.$$

The estimation of integrals using Monte Carlo methods entails assessing the variance in the error of the estimate for a number N of samples. We define the variance $\sigma^2(f)$ for the function $f(x)$ as:

$$\sigma^2(f) = \int dx (f(x) - I)^2.$$

Upon this definition, we can demonstrate the following relation:

$$\int dx_1 \ldots dx_N \left(\frac{1}{N} \sum_{n=1}^{N} f(x_n) - I \right)^2 = \frac{\sigma^2(f)}{N}.$$

This last expression implies that the mean square error of the Monte Carlo estimate is proportional to $\sigma(f)/\sqrt{N}$, where $\sigma(f)$ is the standard deviation of the function f.

Applying the central limit theorem [4], it can be inferred that the probability of our Monte Carlo estimate being within an interval of $I \pm \sigma(f)$ is provided by the limit:

$$\lim_{N \to \infty} P\left(-a\frac{\sigma(f)}{\sqrt{N}} \leq \frac{1}{N} \sum_{n=1}^{N} f(x_n) - I \leq b\frac{\sigma(f)}{\sqrt{N}} \right) = \frac{1}{\sqrt{2\pi}} \int_{-a}^{b} e^{-\frac{t^2}{2}} dt.$$

Additionally, it is important to note that the error in Monte Carlo integration scales inversely with the square root of N, which is independent of the dimension d of the integral. However, the exact variance $\sigma^2(f)$ is often not readily obtainable. Thus, we utilize an estimated variance S^2 instead, computed as:

$$S^2 = \frac{1}{N-1} \sum_{n=1}^{N} (f(x_n) - E)^2 = \frac{1}{N} \sum_{n=1}^{N} f(x_n)^2 - E^2, \tag{S13}$$

where E is the estimated mean of the sampled function values.

Bayesian inference is a method of statistical inference in which the Bayes theorem is used to update the probability of a hypothesis as more evidence or information becomes available. It is a powerful framework for modeling uncertainty in various fields, such as statistics, machine learning, data science, and artificial intelligence.

The core principle of Bayesian inference is encapsulated by the Bayes theorem, which relates the conditional and marginal probabilities of random events. It is given by the formula:

$$P(H|E) = \frac{P(E|H) \cdot P(H)}{P(E)}$$

where:

- $P(H|E)$ is the posterior probability of the hypothesis H given the evidence E;
- $P(E|H)$ is the likelihood of observing the evidence E given that the hypothesis H is true;
- $P(H)$ is the prior probability of the hypothesis H, representing our knowledge or belief about the hypothesis before observing the evidence;
- $P(E)$ is the probability of observing the evidence E, also known as the marginal likelihood or evidence, which acts as a normalizing constant.

The essence of Bayesian inference is to start with a prior belief $P(H)$ about a hypothesis, then update this belief upon observing new evidence E, leading to a posterior belief $P(H|E)$. This process emphasizes the importance of prior knowledge and how it is modified in the light of new data.

In practice, Bayesian inference often involves integrating over many possible parameter values to compute the posterior distribution, which can be computationally challenging. Computational techniques like Markov Chain Monte Carlo (MCMC) methods are commonly employed in this case.

In the context of Bayesian inference, Monte Carlo methods facilitate the estimation of posterior distributions by evaluating integrals of the form

$$I(y) = \int f(y|x)p(x)\,dx.$$

This estimation process is represented by

$$\hat{I}(y) = \frac{1}{n}\sum_{i=1}^{n} f(y|x_i),$$

with each x_i being a sample from the probability distribution $p(x)$.

Now, the standard error (SE) is the standard deviation of its sampling distribution

$$\mathrm{SE}[X] = \frac{\sigma_X}{\sqrt{n}}.$$

So, in our case, using (S13) with $E = \hat{I}(y)$, we can quantify the standard error of this Monte Carlo estimate as

$$\begin{aligned}\mathrm{SE}^2[\hat{I}(y)] &= \frac{1}{n}S^2 \\ &= \frac{1}{n}\left(\frac{1}{n-1}\sum_{i=1}^{n}\left(f(y|x_i) - \hat{I}(y)\right)^2\right)\end{aligned}$$

S3. MCMC Methods

Markov Chain Monte Carlo are a general methods for the simulation of distributions known up to a multiplicative constant. Let v be a σ-finite measure on a state space $(X, \mathcal{X})$ and let $h_\pi \in \mathbb{F}_+(X)$ such that

$$0 < \int_X h_\pi(x)v(dx) < \infty.$$

Typically, X is an open subset of $\mathbb{R}^d$ and v is the Lebesgue measure, or X is countable and v is the counting measure. This function is associated to a probability measure π on X defined by

$$\pi(A) := \frac{\int_A h_\pi(x)v(dx)}{\int_X h_\pi(x)v(dx)}$$

We want to approximate expectations of functions $f \in \mathbb{F}_+(X)$ with respect to π

$$\pi(f) = \frac{\int_X f(x) h_\pi(x) v(dx)}{\int_X h_\pi(x) v(dx)}$$

If the state space X is high-dimensional and h_π is complex, direct numerical integration is not an option. The classical Monte Carlo solution to this problem is to simulate i.i.d. random variables $Z_0, Z_1, \ldots, Z_{n-1}$ with distribution π and then estimate $\pi(f)$ by the sample mean

$$\hat{\pi}(f) = n^{-1} \sum_{i=0}^{n-1} f(Z_i).$$

This gives an unbiased estimate with a standard deviation of order $O\left(n^{-1/2}\right)$ provided that $\pi\left(f^2\right) < \infty$. Furthermore, by the central limit theorem, the normalized error $\sqrt{n}(\hat{\pi}(f) - \pi(f))$ has a limiting normal distribution, so that confidence intervals are easily obtained [5, Chapter 6].

The problem often encountered in applications is that it might be very difficult to simulate i.i.d. random variables with distribution π. Instead, the Markov Chain Monte Carlo (MCMC) solution is to construct a Markov chain on X that has π as invariant probability. The hope is that regardless of the initial distribution ξ, the law of large numbers will hold, i.e.,

$$\lim_{n \to \infty} n^{-1} \sum_{k=0}^{n-1} f(X_k) = \pi(f) P_\xi.$$

At first sight, it may seem even more difficult to find such a Markov chain than to estimate $\pi(f)$ directly. In the following subsections, we will exhibit several such constructions.

S4. Metropolis-Hastings

Let Q be a Markov kernel having a density q with respect to v, i.e.,

$$Q(x, A) = \int_A q(x, y) v(dy)$$

for every $x \in X$ and $A \in \mathcal{X}$.

The Metropolis-Hastings algorithm [6, Section 2.3.1], also described in Figure S1, proceeds in the following way. An initial starting value X_0 is chosen. Given X_k, a candidate move Y_{k+1} is sampled from $Q(X_k, \cdot)$. With probability $\alpha(X_k, Y_{k+1})$, it is accepted, and the chain moves to $X_{k+1} = Y_{k+1}$. Otherwise, the move is rejected, and the chain remains at $X_{k+1} = X_k$. The probability $\alpha(X_k, Y_{k+1})$ of accepting the move is given by

$$\alpha(x, y) = \begin{cases} \min\left(\frac{h_\pi(y)}{h_\pi(x)} \frac{q(y,x)}{q(x,y)}, 1\right) & \text{if } h_\pi(x) q(x, y) > 0 \\ 1 & \text{if } h_\pi(x) q(x, y) = 0 \end{cases} \tag{S14}$$

The acceptance probability $\alpha(x, y)$ only depends on the ratio $h_\pi(y)/h_\pi(x)$; therefore, we only need to know h_π up to a normalizing constant. In Bayesian inference, this property plays a crucial role.

This procedure generates a Markov chain,

$$\mathbb{X} = \{X_k, k \in \mathbb{N}\}, \tag{S15}$$

with a Markov kernel P given by

$$P(x, A) = \int_A \alpha(x, y) q(x, y) v(dy) + \bar{\alpha}(x) \delta_x(A)$$

with

$$\bar{\alpha}(x) = \int_X \{1 - \alpha(x,y)\} q(x,y) v(dy)$$

and $\delta_x(A)$ is the measure of A. The quantity $\bar{\alpha}(x)$ is the probability of remaining at the same point.

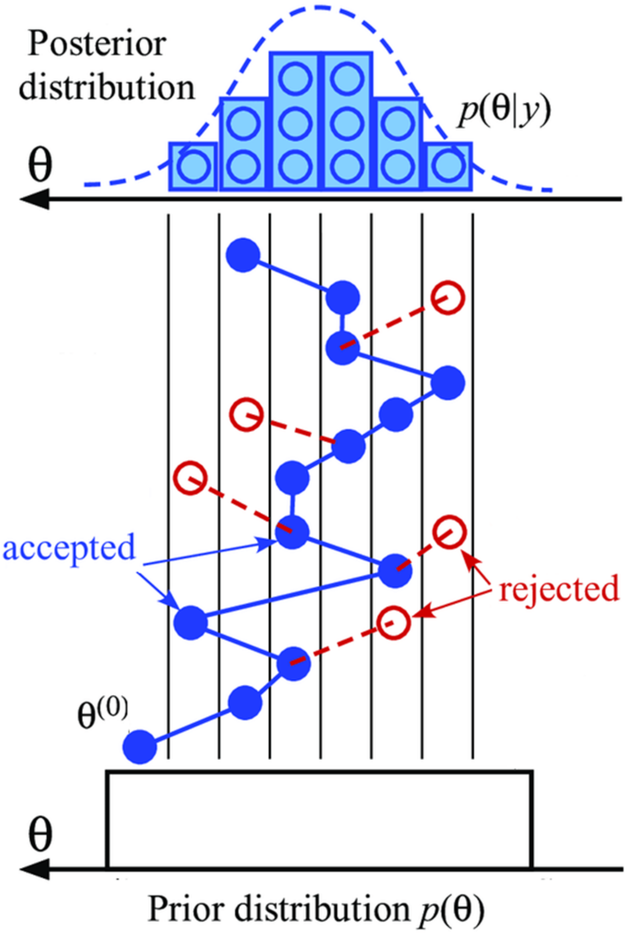

Figure S1. Representation of a typical Metropolis-Hastings algorithm. The bottom part shows the prior distribution $p(\theta)$ with the initial point $\theta^{(0)}$. The algorithm proposes moves, shown as blue (accepted) and red (rejected) circles. Accepted moves (solid blue arrows) contribute to the Markov chain, while rejected moves (dashed red arrows) are ignored. The top part shows the posterior distribution $p(\theta|y)$ as a curve and a histogram, representing the target distribution. This process demonstrates how the algorithm samples from the posterior distribution by iteratively proposing and accepting or rejecting moves.[8]

S5. Differential Evolution

Differential evolution method is one example of an evolutionary algorithm. We will see its use to create an MCMC algorithm, combined with Metropolis-Hastings. In fact, it can be used to optimize the "jumps" taken in the construction of the Markov chain.

Evolutionary Algorithms (EA) are population-based metaheuristics with a track record of outperforming traditional approaches in situations where conventional methods can not be used. Inspired by Darwin's evolutionary theory, these algorithms created the Evolutionary Computation [9]. This field is dedicated to digitally replicating the evolutionary process.

Algorithm S1 Metropolis-Hastings Algorithm [7]

Initialize $x^{(0)}$
for $i = 0$ to $N - 1$ **do**
  Generate a candidate x' from a proposal distribution $q(x'|x^{(i)})$
  Calculate acceptance ratio $\alpha = \frac{p(x')q(x^{(i)}|x')}{p(x^{(i)})q(x'|x^{(i)})}$
  Generate a uniform random number $u \sim \text{Uniform}(0, 1)$
  if $u \leq \min(1, \alpha)$ **then**
    Accept the candidate: $x^{(i+1)} = x'$
  else
    Reject the candidate: $x^{(i+1)} = x^{(i)}$
  end if
end for

The emergence of EA was in the 1960s and can be characterized by three primary movements: genetic algorithms, evolutionary strategies, and evolutionary programming. By the early 1990s, a fourth trend, genetic programming, became part of it:

- *Genetic Algorithms* - The concept, which finds its origins in the work of J. Holland in 1975 [10], translates the mechanics of biological evolution into a computational paradigm. Simulating biological adaptation in a digital format. Later, K. DeJong [11] formalized genetic algorithms for optimizing binary search spaces. Moreover, D. Goldberg [12], significantly improved the availability and efficiency of these algorithms;
- *Evolution Strategies* - They come from the works of I. Rechenberg [13] and H. Schwefel [14], who were trying to solve an aerodynamic engineering challenge. They proposed an innovative concept for optimization beyond the conventional methods, focusing on the principles of adaptation and self-modulation, which are core to evolutionary algorithms;
- *Evolutionary Programming* - It is a concept developed by L.J. Fogel [15,16]. Its objective was to simulate evolutionary processes to solve complex problems. This led to a new form of machine intelligence—evolutionary machines, that were not only efficient optimizers but also capable of self-improvement;
- *Genetic Programming* - J. Koza [17] introduced this strategy, a significant advancement in the evolution of complex computational structures. This method utilized the framework of tree structures, similar to neural networks, to encode solutions as expressions. Each node within these graphs was associated with specific operations that addressed elements of the given problem.

Evolutionary Algorithms are characterized by a vocabulary specific to the domain. The starting point is a set of potential solutions, referred to as a population. Each individual within this population represents a possible solution and is an entity known as an individual. In the absence of preexisting optimal solutions, Evolutionary Algorithms begin by initializing a diverse population. This initial setup is called *initialization*.

Following this, each individual is evaluated to determine its fitness for solving the problem at hand, known as *evaluation*.

Once the initial assessment is complete, the *evolutionary cycle* begins. This involves a series of iterations, or generations, where selection, reproduction, and variation occur.

We can represent every evolutionary cycle in three steps:

1. *Selection* - Choose individuals from the population that are most likely to successfully reproduce;
2. *Variations* - Introduce random changes to the selected individuals, primarily through two mechanisms: crossover and mutation. These processes create *"children"* from the chosen *"parents"*;
3. *Replacement* - Update the population for the next generation, typically by selecting the fittest individuals from both the "*parents*" and the *"children"*.

Algorithm S2 Typical Evolutionary Algorithm (EA) [18]

Generation $g \leftarrow 0$
Population $P^g \leftarrow$ INITIALIZE
Fitness $f(P^g) \leftarrow$ EVALUATE
while (**not** stopping condition) **do**
    // proceed to the next evolutionary cycle //
    $g \leftarrow g + 1$
    Parents $\leftarrow$ SELECT(P^g)
    Children $\leftarrow$ VARY(Parents (Crossover, Mutation, ...))
    Fitness $\leftarrow$ EVALUATE(Children)
    Replacement $P^g \leftarrow$ SURVIVE(Parents and Children)
end while

The setting of Differential Evolution (DE) is a standard optimization problem.

Let $\emptyset \neq M \subset \mathbb{R}^D$, we consider a function $f : M \to \mathbb{R}$, which we call our *objective function*. This could also be known as a fitness or cost function.
The main goal is to find $x^* \in M$, the minimum point of f, i.e., to find M such that

$$\text{for any } X \in M : f(X) \geq f(X^*) = f^*,$$

where f^* is called a global minimum of f.

In our case, we have that M is in the following form, with $\mathbf{X} = \{x_1, \ldots, x_D\} \in \mathbb{R}^D$.

$$M = \{\mathbf{X} \mid g_k(\mathbf{X}) \leq 0, l_j \leq x_j \leq h_j \text{ with } k \in \{1, \ldots, m\}, j \in \{1, \ldots, D\}\},$$

representing a problem with g_k's as constraints and l_j, h_j, respectively, the lower and upper bounds.

Algorithm S3 Differential Evolution (DE) [18]

Require: F, Cr, N, P – control parameters
Initialize $P^0 \leftarrow \{x_1, \ldots, x_{NP}\}$
Evaluate $f(P^0) \leftarrow \{f(x_1), \ldots, f(x_{NP})\}$
while (not stopping condition) **do**
    for all $x \in P^G$ **do**
        $P^G \rightarrow \pi = \{\xi_1, \xi_2, \ldots, \xi_n\}$
        $v \leftarrow$ Differentiation$(\pi, F, \text{Strategy})$
        $v \leftarrow$ Crossover(v, Cr)
        $x \leftarrow$ Selection(v, x)
    end for
    $G \leftarrow G + 1$
end while

The preceding algorithm processes (with $n = 3$):

- *Differentiation* - as is shown in Figure S2, for each target vector $\{x_1^G, \ldots, x_{NP}^G\}$, we select 3 random indexes $r_1, r_2, r_3 \in \{1, \ldots, NP\}$, and we obtain the "mutant" vector

$$v_i^{G+1} = x_{r_1}^G + F \cdot (x_{r_2}^G - x_{r_3}^G);$$

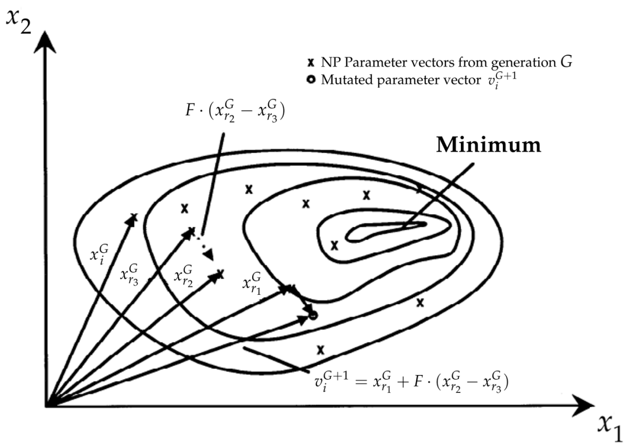

Figure S2. An example of a two-dimensional cost function showing its contour lines and the process for generating v_i^{G+1}. [19]

- *Crossover* - as is shown in Figure S3, in order to increase the diversity of the perturbed parameter vector. We create this vector

$$u_i^{G+1} = (u_{1i}^{G+1}, u_{2i}^{G+1}, \ldots, u_{Di}^{G+1})$$

with,

$$u_{ji}^{G+1} = \begin{cases} v_{ji}^{G+1} & \text{if } rand_j \leq Cr \text{ or } j = rand_i \\ x_{ji}^G & \text{otherwise,} \end{cases}$$

where $j = 1, \ldots, D$, $rand_j$ is the j-th evaluation of a uniform random number generator in $[0, 1]$ and $Cr \in [0, 1]$ represents the constant crossover. The condition "$j = rand_i$" is to make sure that u_{ji}^{G+1} gets at least one parameter from v_{ji}^{G+1};

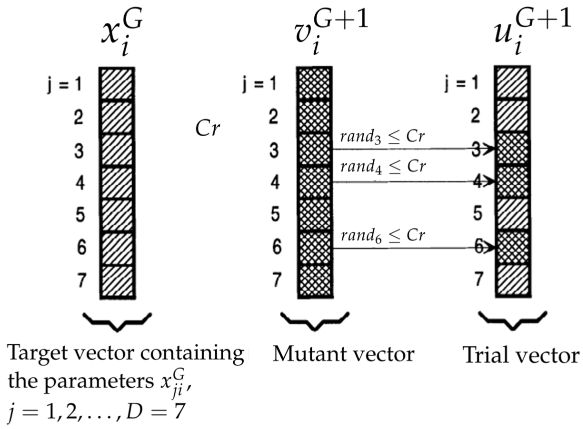

Figure S3. Crossover process for $D = 7$ parameters. [19]

- *Selection* - here we decide if the trial vector should become a member of generation $G + 1$. The trial vector u_i^{G+1} is compared to the target vector x_i^G using the greedy criterion: if the vector u_i^{G+1} has a smaller cost function value than x_i^G, then x_i^{G+1} is set to u_i^{G+1}; otherwise, the old value is retained.

References

1. Lindelöf, E. Sur l'application de la méthode des approximations successives aux équations différentielles ordinaires du premier ordre. *Comptes Rendus Hebdomadaires Des Séances De L'Académie Des Sciences*. **116**, 454-457 (1894)
2. Hartman, P. Ordinary Differential Equations: Second Edition. (Society for Industrial,1982)
3. Weinzierl, S. *Introduction to Monte Carlo Methods*; Springer: Berlin/Heidelberg, Germany, 2000.
4. Dekking, F. A Modern Introduction to Probability and Statistics: Understanding why and how. (Springer Science & Business Media,2005)
5. Robert, C. & Casella, G. Monte Carlo Statistical Methods. (Springer New York,2004), https://doi.org/10.1007/978-1-4757-4145-2, Hardcover ISBN: 978-0-387-21239-5, Softcover ISBN: 978-1-4419-1939-7, eBook ISBN: 978-1-4757-4145-2
6. Douc, R., Moulines, E., Priouret, P. & Soulier, P. Markov chains. (Springer,2018)
7. Chib, S. & Greenberg, E. Understanding the metropolis-hastings algorithm. *The American Statistician*. **49**, 327-335 (1995)
8. Tomic, S., Beko, M., Camarinha-Matos, L. & Oliveira, L. Distributed Localization with Complemented RSS and AOA Measurements: Theory and Methods. *Applied Sciences*. **10** pp. 272 (2019,12)
9. Spears, W., De Jong, K., Bäck, T., Fogel, D. & De Garis, H. An overview of evolutionary computation. *European Conference On Machine Learning*. pp. 442-459 (1993)
10. Holland, J. Adaptation in natural and artificial systems. an introductory analysis with applications to biology, control and artificial intelligence. *Ann Arbor: University Of Michigan Press*. (1975)
11. De Jong, K. An analysis of the behavior of a class of genetic adaptive systems.. (University of Michigan,1975)
12. Goldberg, D. & Holland, J. Genetic Algorithms and Machine Learning. *Machine Learning*. **3**, 95-99 (1988,10), https://doi.org/10.1023/A:1022602019183
13. Rechenberg, I. Evolutionsstrategie: Optimierung technischer Systeme nach Prinzipien der biologischen Evolution. (Frommann-Holzboog,1973)
14. Schwefel, H. Numerical Optimization of Computer Models. (Wiley,1981)
15. Fogel, L., Owens, A. & Walsh, M. Artificial intelligence through simulated evolution. (Wiley,1966)
16. Fogel, D. Evolving artificial intelligence. (University of California at San Diego,1992)
17. Koza, J. Genetic Programming: On the Programming of Computers by Means of Natural Selection. (Bradford,1992)
18. Feoktistov, V. Differential evolution. (Springer,2006)
19. Storn, R. & Price, K. Differential evolution–a simple and efficient heuristic for global optimization over continuous spaces. *Journal Of Global Optimization*. **11** pp. 341-359 (1997)
